# Medical Society of the County of Albany, Semi-Annual Meeting June 10th

**Published:** 1873-07

**Authors:** F. C. Curtis

**Affiliations:** Secretary


ART. IV.—*Medical Society of the County of Albany. Semi-Annual Meeting June* 10*th*.* Reported by F. C. CURTIS, M. D., Secretary.

The semi-annual meeting of the society was held on June 10th, at 3 P. M., in the Supervisor's room, City Hall.

Dr. VAN DERVEER, President, in the chair.

After the minutes of the last meeting had been read and approved, the President made the following remarks:

As our society increases in members, the number of those whom death take from us must necessarily become greater, and for the first half of our present year we have been called to mourn the loss of an unusual number. To-day I have to announce to you the death of Dr. John H. Becker, who died of inflammation of the lungs, June 1st, in the 45th year of his age. He had long been a member of the society, but from his location in the country, where he was engaged in a laborious practice, he never became an active member; seldom meeting with the society, and to many of the younger members was a comparative stranger. I would suggest that suitable resolutions be presented and adopted by the society.

As our semi-monthly meetings have ended, I desire at this time to make some acknowledgment for the prompt and efficient manner in which you have given support to these meetings.

We have held in all ten semi-monthly and two special meetings. These meetings have been uniformly well attended.

There have been presented seventeen regularly prepared papers, besides a good number of pathological specimens. Also two important discussions have occupied the attention of the Society.

In behalf of the officers I desire to render to you our heartfelt thanks for the generous support you have given us thus far to elevate our profession, and to place our society among the first and foremost in the State.

Dr. S. H. FREEMAN then said: Mr. President and gentlemen of the society—My earliest associations with Dr. Becker were of peculiar interest. He becan the study of medicine in our office

* Owing to lack of space the report of the proceedings for May 28th was laid over until next mouth.

soon after I began the practice, and at an early period of my partnership with Dr. Armsby. He was a young man of promise, and the favored son of liberal and indulgent parents. When nearly prepared for graduation he, unadvisedly, went to California during the period of the gold excitement, with two older fellow students from our office who had just graduated—Dr. Tabor, of this city, and Dr. Steele—both of whom have since died. After an absence of about two years he returned and graduated, with credit to himself, and entered upon the practice of medicine in his native town of New Scotland, where he continued to reside until his death.

In his student life and in his early professional career I felt a deep interest, and was frequently gratified by repeated evidences of his professional skill and success. Of late years I have known but little of his personal history, but have understood that his tastes inclined him to partially relinquish his medical practice for the less arduous pursuits of agricultural life. As a tribute to his memory, and as an expression of our sympathy for his family, I offer the following resolutions:

*Resolved*, That we record our deep sorrow at the death of our professional brother, John H. Becker, so unexpectedly summoned into eternity, as he was just entering upon the meridian of life.

*Resolved*, That whilst we bow in submission to the Divine will in this afflictive dispensation, we find a new incentive to our own fidelity to God and to humanity.

*Resolved*, That we sincerely sympathize with the sorrowing family in their bereavement, and that we transmit a copy of these resolutions to them. Unanimously adopted.

Dr. James S. Bailey, chairman of the committee on publication, presented the report, which, after much discussion, in which Drs. Craig, Porter, Northrop, Swinburn, Quackenbush, and McNaughton took part, was, on motion of Dr. Craig, accepted, and referred back to the committee for further consideration.

Dr. James P. Boyd, chairman of the board of censors, presented the following names for admission into the society : James N. Haines, Alma S. Allen, J. H. Blatner, James S. Hill, G. A. Jones, J. H. Hannan, James P. Boyd, Jr., J. Emerson Allen, of Scranton, Pa., Frank Garbutt and E. A. Green. On motion these gentlemen were admitted to membership on compliance with the by-laws.

Dr. A. W. Shiland, of West Troy, vice-president of the society, then delivered the following semi-anuual address:

Gentlemen of the Society: In ancient times the theory and practice of medicine was mingled with the wildest absurdities. We learn from ancient mythology how Epimethius carelessly raised the lid from Pandora's box, and all the plagues that since affect our race came from that infernal source.

The incantations of necromancy were formerly considered the most powerful means of curing disease. The mysteries of astrology were invoked, and it was firmly believed that sidereal conjunctions, or planetary influences, determined the life or death of every human being.

But the delusions of ancient mythology and superstition have nearly passed away, and even the advocates of the late Hahneman system no longer shake their infinitesimal dilutions with the vain hope of increasing their potency, neither do they employ decimal ciphers to indicate the medicinal particle, too infinitely small for microscopical observation.

It is a common observation that there has of late years been great progress in every art that appertains to the elevation of humanity, and this, I may say, is especially true of the healing art. There is a common bond and relationship between the science of medicine and all the liberal arts. A thorough knowledge of medicine implies an acquaintance with every other department of science. It is our province to investigate the different departments of animate and inanimate nature. The elements of the material universe afford subjects for our most profound study. Their affinities and relations, so infinitely varied, must be well understood by the intelligent physican.

He must know also the laws that govern the mental, the vital principle that animates the physical world, that mysterious unity of body and mind which we are constantly endeavoring to maintain and harmonize in the humanity that peoples the earth. It should be our constant endeavor to elevate our profession and maintain the highest rank in the advancement of civilization. The qualifications of a student of medicine are, for the most part, a matter of too little consideration. A good primary education is deemed most essential to the student of law or theology, and why should it be considered any less important to the student of medicine? Why should we be less faithless to our own calling? Why should young men be encouraged to enter our profession who have no fitness for it? Intelligence virtue and honor should constitute the passport to our profession. Second to no other avocation in importance, the profession and science of medicine will ever maintain the highest rank in every enlightened community.

The intelligent and honorable physician will supersede every unworthy pretender. The interests and welfare of humanity demand the highest educational attainments in those who practice the healing art.

The frequent convocations of this society are intended to promote this object and create a sentiment in favor of the highest culture.

I would gladly improve every opportunity to meet my brothers in the profession here, and the generous consideration which you have shown me, will ever stimulate me to greater effort to prove myself worthy of your highest appreciation.

Whatever progress I may have made in the art of healing, I need hardly tell you I have sadly neglected the art of speaking, and I ask your kind indulgence while I attempt, on this occasion, to eulogize our most noble profession and exalt the sublime art we practice.

The healing art, the art divine, to give relief from pain; to soothe the dying or light the lamp of life that dimly burns; to give the bloom of health where pale disease has set its seal; to restore the bleeding body, torn or crushed by the many accidents of life, and while wrecked with mortal agony to soothe to oblivion with ethers balmy inspiration, no human art can this excel. Body and mind are bound by such mysterious, sympathetic ties, that none but those who know our art can tell the laws that rule the physical and mental being. With constant care we guard this mortal and immortal body and mind, mysterious brotherhood in strange communion joined, from youth to age, from birth to dissolution. What nobler work! To heal the mind diseased; to restore lost reason; to cure the maniac fiend, and make him a noble, God-like man. Mind, mind alone, is life, and light, and power; the body dies, but not the soul that springs immortal there.

Our heavenly art makes these agree
And act in perfect harmony,
'Till soul has reached its destiny,
And flesh resolves to earth again.

Dr. FREEMAN moved a vote of thanks to Dr. Shiland for his address. Adopted.

Dr. JAMES S. BAILEY said that there were several names in the directory of members who had not paid their yearly dues, and offered a resolution.

*Resolved*, That any member of the Albany Medical Society who shall be in arrears for two years in paying his yearly dues is not entitled to suffrage in this society, nor shall he be hereafter reinstated uuless back dues are paid, or without an unanimous vote of the society.

*Resolved*, That the Secretary obtain the names of such delinquents from the Treasurer and drop their names from the roll. Adopted.

The President then said:

I would announce the following names as composing the committee called for by Dr. James S. Bailey's resolution regarding the proposed .entertainment to be given to Dr. Peter P. Staats: Drs. James S. Bailey, James McNaughton, P. V. Quackenbush, S. H. Freeman, John Swinburne, Levi Moore, F. C. Curtis.

Dr. J. M. BIGELOW then complimented the society on the success of their semi-monthly meetings, and offered the following resolution:

*Resolved*, That the thanks of the society are due to Drs. J. S. Bailey and F. Curtis for the reports they had made of the several proceedings and for having them inserted in the Philadelphia and Buffalo Medical Journals, and also to the press of this city for their kindness in noticing and reporting their meetings. Adopted.

On motion of Dr. Craig the meeting then adjourned.

